# Emerging Fungal Pathogen Rhodotorula Species Isolated From a Patient With a Lung Malignancy

**DOI:** 10.7759/cureus.58131

**Published:** 2024-04-12

**Authors:** William Wheeler, Christopher Clark, Stephen DiGiuseppe

**Affiliations:** 1 Osteopathic Manipulative Medicine, Edward Via College of Osteopathic Medicine, Monroe, USA; 2 Microbiology, Edward Via College of Osteopathic Medicine, Monroe, USA

**Keywords:** nosocomial and opportunistic infections, commensal, emerging yeast, fungal lung infection, b-cell lymphoma, opportunistic fungal infection

## Abstract

*Rhodotorula *is a genus of ubiquitous pigmented yeast found in the environment and as a commensal of human and animal microbiota. Previously considered nonpathogenic, *Rhodotorula* has emerged as an important cause of nosocomial and opportunistic infections in susceptible patients. While *Rhodotorula* spp. are common commensals in healthy individuals, the yeast may overgrow in patients with compromised immune systems causing disease. Herein, we provide a detailed presentation of a rare case involving a 79-year-old Caucasian female with a lung malignancy who developed massive cavitations in her lungs. The patient's lung tissue was cultured and grew an unidentified species of the genus *Rhodotorula*. The patient's health declined rapidly, and she expired due to hypoxemia. Clinicians must recognize patient groups potentially at risk for infection with *Rhodotorula* spp. Early identification and initiation of appropriate interventions are crucial in reducing mortality associated with this opportunistic fungal infection.

## Introduction

*Rhodotorula *spp. are common yeasts found in environmental sources such as air, soil, lakes, ocean water, milk, and fruit juice. *Rhodotorula *spp. often produce red, orange, or pink colonies, which grow on shower curtains, bathtubs, toilet bowls, and toothbrushes [1]. As a commensal of human and animal microbiota, *Rhodotorula *spp. colonize the skin, nails, and gastrointestinal, urinary, and respiratory tracts [2]. A meta-analysis determined that the most common infections of *Rhodotorula *spp. were involving the bloodstream, central nervous system (CNS), ocular infections, and peritoneal dialysis-associated peritonitis [3]. A review of reported cases determined that the most common risk factor was related to central venous catheters in association with more aggressive treatment modalities. These modalities included admission to intensive care units, short- and long-term parenteral nutrition, broad-spectrum antibiotics, organ transplants, and chemotherapy [1,4]. Currently, *Rhodotorula *infections occur most often in immunocompromised patients with hematologic cancers, while AIDS, chronic renal failure, cirrhosis, and gastrointestinal disorders were also considered factors [1]. Herein, we describe a case with *Rhodotorula *sp. overgrowth in a woman who presented with B-cell lymphoma.

## Case presentation

A 79-year-old Caucasian female patient presented to the emergency room (ER) with chest pain that started three days prior to admission. The patient reported that she was scheduled for a lung biopsy that morning after a lung mass was found during a previous visit to the ER. She was tachycardic (146 bpm) and hypotensive (99/62 mm Hg), consistent with atrial fibrillation with a rapid ventricular response. Past medical history was significant for arthritis, hyperlipidemia, mitral valve prolapses, and cataract surgery. She denies any past stent placement, myocardial infarctions, or deep venous thrombosis. Her troponin level was normal. Physical examination was unremarkable. She showed an increased white blood cell (WBC) count of 15,700 per μL, with increased neutrophils at 14,700 per μL. She had high levels of blood urea nitrogen (BUN) (21 mg/dL) and low levels of creatinine (0.47 mg/dL). Her liver enzymes were elevated as well, despite the absence of alcohol use or any medications known to affect the liver. Her alanine transaminase (ALT) and aspartate aminotransferase (AST) levels were 92 units/L and 97 units/L, respectively. Also, her initial X-ray and CT scan showed a mass in the right lung but no other pulmonary infiltrate. The patient was given intravenous fluids for possible dehydration and intravenous metoprolol to control her heart rate. She was hospitalized for further management.

**Figure 1:**
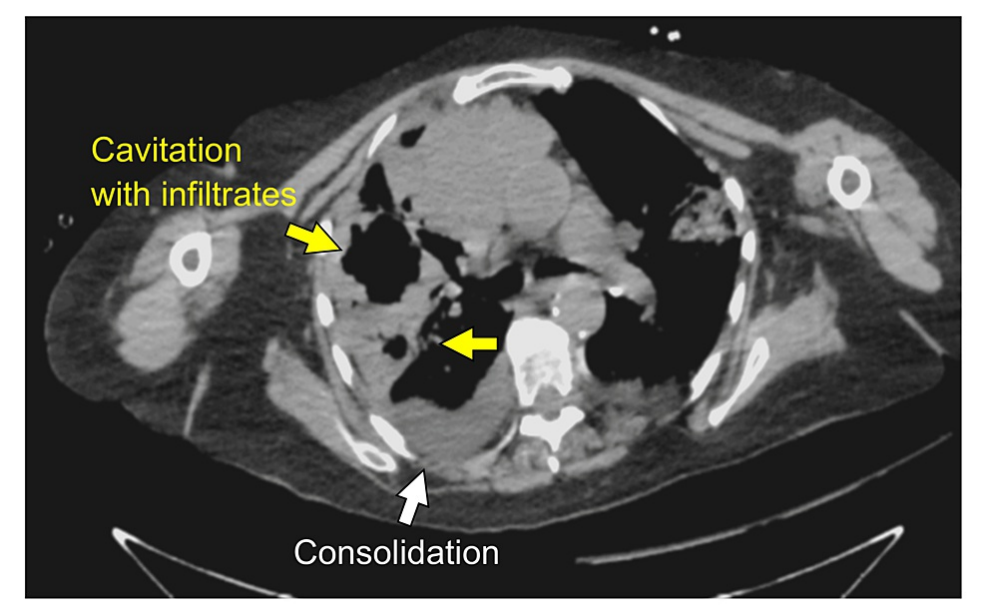
CT scan of the patient's thorax. CT scan of the patient's thorax showed cavitation in the right lung, which increased in volume. Other findings include bibasilar atelectasis, consolidation, ground-glass opacification, and infiltrates.

Upon hospitalization, the patient's lung mass was biopsied, and an infectious disease specialist was consulted. Repeat X-rays showed increased infiltrates, indicating the development of pneumonia. She was treated for sepsis because the patient met both systemic inflammatory response syndrome (SIRS) criteria and quick sequential organ failure assessment (qSOFA) with elevated WBC count (15,700 per μL), respiration (22 rpm), and heart rate (120 bpm) resulting in the diagnosis of sepsis secondary to pneumonia [5-7]. Piperacillin/tazobactam and vancomycin were started. Over the next week, she also had a bone marrow biopsy and had to be placed on supplemental oxygen due to hypoxemia. As her health declined, the decision was made to undergo a bronchoscopy and place a chest tube to help her remove secretions. Lung samples were obtained for lab culturing. The biopsy results were returned, and the patient was diagnosed with B-cell lymphoma. She was placed in the critical care unit and was given 55 L of oxygen for worsening hypoxemia. The patient's condition continued to decline, and a repeat CT scan showed massive cavitations and necrosis along with ground-glass appearance and consolidated infiltrates in both upper lobes of the lungs (Figure 1). The pace of cavitation enlargement far exceeded what was expected of common bacterial infections in immunocompromised patients. Lung biopsies and cultures had yet to grow any bacteria or fungi. Meropenem and doxycycline were added to bacterial coverage, and isavuconazole was added for fungal coverage. Though broad-spectrum antibiotic coverage was started, her pulmonary status did not improve. Oxygen was increased from 55 to 80 L over the next week. Cultures from biopsies were still negative. Eventually, the patient was moved to a long-term care facility for ongoing antibiotic and antifungal treatments.

In long-term care, the patient was followed up by an oncologist and placed under palliative care. Unfortunately, she eventually expired secondary to complications of infection. Less than one week prior to her death, the lung samples grew *Rhodotorula *spp., but at the time of this discovery, the patient refused additional intervention.

## Discussion

*Rhodotorula *spp. produce distinctive orange, pink, or red colonies when grown on Sabouraud's dextrose agar with optimal growth temperature between 18°C and 22°C [8]. To confirm the identity of a suspected isolate, genomic DNA is extracted, and the internal transcribed spacer (ITS) regions are amplified with universal ITS primers followed by sequencing and the basic local alignment search tool (BLAST) [9]. *Rhodotorula *spp. have been isolated from a variety of clinical sources including the blood, skin, vagina, and respiratory tract. The incidence of pneumonia caused by *Rhodotorula *spp. is unknown. The mycology reference laboratory in Spain identified two out of 29 isolates were from respiratory tract samples [10]. Another study reports that *Rhodotorula *represent up to 0.5% fungemia in the United States [11]. *Rhodotorula *fungemia frequently occurs in patients with blood or bone marrow cancers and those with some form of catheter. In contrast, half of these infections can occur due to breakthrough infections after receiving previous antifungals. *Rhodotorula *infections can cause intra-abdominal infections, abscesses, and pneumonia, which can result in sepsis and is the most direct cause of death in patients with disseminated *Rhodotorula *infections [12,13].

Treatment of *Rhodotorula *spp. is complex because the cell wall does not typically contain 1,3-β-glucan, rendering them intrinsically resistant to echinocandins [8]. A study analyzing susceptibility concluded that fluconazole, itraconazole, and voriconazole were inactive against the majority of isolates, while amphotericin B and flucytosine exhibited good antifungal activity [10]. The patient was treated with isavuconazole, which may have limited antifungal activity against *Rhodotorula *isolates based on conflicting reports [14,15]. More studies are needed to investigate the susceptibility of antifungals against clinical *Rhodotorula* isolates. Nevertheless, the current recommended treatment for *Rhodotorula *involves aggressive treatment with amphotericin B and the immediate removal of a catheter if present [8,16].

## Conclusions

*Rhodotorula *sp. is an environmental fungus that can be transmitted to humans. Overgrowth of *Rhodotorula *spp. are typically suppressed in healthy individuals; however, these fungi have emerged as opportunistic pathogens in immunocompromised hosts. This case provides a detailed presentation of a patient with a lung malignancy who succumbed to a *Rhodotorula *sp. infection in the lung. Identification of the *Rhodotorula *spp. must be prompt because azole use in such patients delays early effective amphotericin B treatment. A significant risk factor for *Rhodotorula *spp. colonization is the catheterization of cancer patients, and clinicians must be aware of this fungus to avoid delaying lifesaving treatment. 
